# Characterization of a cold-active and salt tolerant esterase identified by functional screening of Arctic metagenomic libraries

**DOI:** 10.1186/s12858-016-0057-x

**Published:** 2016-01-19

**Authors:** Concetta De Santi, Bjørn Altermark, Marcin Miroslaw Pierechod, Luca Ambrosino, Donatella de Pascale, Nils-Peder Willassen

**Affiliations:** NorStruct, Department of Chemistry, Faculty of Science and Technology, UiT The Arctic University of Norway, Tromsø, Norway; Institute of Protein Biochemistry, National Research Council, Naples, Italy

**Keywords:** Metagenomics libraries, Cold-active esterase, Salt, Homology modeling, Biotechnological applications

## Abstract

**Background:**

The use of metagenomics in enzyme discovery constitutes a powerful approach to access to genomes of unculturable community of microorganisms and isolate novel valuable biocatalysts for use in a wide range of biotechnological and pharmaceutical fields.

**Results:**

Here we present a novel esterase gene (*lip3*) identified by functional screening of three fosmid metagenomic libraries, constructed from three marine sediment samples. The sequenced positive fosmid revealed an enzyme of 281 amino acids with similarity to class 3 lipases. The 3D modeling of Lip3 was generated by homology modeling on the basis of four lipases templates [PDB ID: 3O0D, 3NGM, 3G7N, 2QUB] to unravel structural features of this novel enzyme. The catalytic triad of Lip3 was predicted to be Asp207, His267 and the catalytic nucleophile Ser150 in a conserved pentapeptide (GXSXG). The 3D model highlighted the presence of a one-helix lid able to regulate the access of the substrate to the active site when the enzyme binds a hydrophobic interface. Moreover an analysis of the external surface of Lip3 model showed that the majority of the surface regions were hydrophobic (59.6 %) compared with homologous lipases (around 35 %) used as templates. The recombinant Lip3 esterase, expressed and purified from *Escherichia coli*, preferentially hydrolyzed short and medium length *p*-nitrophenyl esters with the best substrate being *p*-nitrophenyl acetate. Further characterization revealed a temperature optimum of 35 °C and a pH optimum of 8.0. Lip3 exhibits a broad temperature stability range and tolerates the presence of DTT, EDTA, PMSF, β-mercaptoethanol and high concentrations of salt. The enzyme was also highly activated by NaCl.

**Conclusions:**

The biochemical characterization and homology model reveals a novel esterase originating from the marine Arctic metagenomics libraries with features of a cold-active, relatively thermostable and highly halotolerant enzyme. Taken together, these results suggest that this esterase could be a highly valuable candidate for biotechnological applications such as organic synthesis reactions and cheese ripening processes.

**Electronic supplementary material:**

The online version of this article (doi:10.1186/s12858-016-0057-x) contains supplementary material, which is available to authorized users.

## Background

Extreme environments represent a great microbial resource for novel enzymes, the majority of which remains to be discovered. Metagenomics, the technique to access the genome content resource of non-cultivated microbes, is a powerful tool used in the discovery of novel industrial enzymes for biotechnological and pharmaceutical applications [1–5]. More than 99 % of the microorganisms cannot be cultivated [6] but discovered, using an alternative metagenomics approach to the traditional microbial screening methods to isolate enzyme from extreme environments [7–9].

Based on the direct cloning of the metagenomic DNA [10] for the construction of large clone libraries, metagenomics gives access to new genes, complete pathways and their products by multiple screening methods. Despite that there are several limitations in screening of such libraries, such as the functional expression of genes in a heterologous, screening host, the metagenome-approach has led to the discovery of many novel enzymes such as new esterases (carboxyl ester hydrolases, EC 3.1.1.1) and lipases (triacylglycerol lipases, EC 3.1.1.3) [11, 12]. Lipolytic enzymes are found in all living organisms and most of the commercially produced enzymes originate from microbial sources. Lipolytic enzymes can be grouped into 8 different families based on their sequence, structure and biological functions [13]. These enzyme families are all characterized by a catalytic triad consisting of a nucleophilic serine, a catalytic acid (aspartate or glutamate) and a histidine residue which is located in a conserved Gly-Xaa-Ser-Xaa-Gly pentapeptide that forms a sharp elbow in the center of the α/β-fold [14]. Lipases can be distinguished from esterases by exhibiting the interfacial activation [15]. Both enzymes have a secondary structural elements, called lids, that change conformation to accommodate the substrates [16, 17]. This lid moves to expose the catalytic cleft at the lipid-water interface according to the activation mechanism typical of lipases. However, there are exceptions such as the previously characterized *Candida antarctica* Lip B [18].

To date, numerous novel lipolytic enzymes have been identified by functional metagenomics analysis of various microbial habitats, such as soil [19–21], hot springs [22], lake water [23] and marine sediments [24, 25]. In particular, cold-active esterases and lipases have been studied because of their structural flexibility if compared to mesophilic and thermophilic counterparts. A reduced number of disulfide bridges and prolines in loop structure has been observed in several cold lipases with a high catalytic activity and stability at low temperatures. Thus, lipolytic enzymes have emerged as key enzymes in the growing biotechnology industry [26].

In this study, we screened three small metagenomic libraries constructed from marine sediment samples in order to identify new esterases for developing a cocktail, together with other lypolitic enzymes, with application in food industry [27, 28]. After sequencing of a positive clone, we found the gene responsible for the esterase activity seen on tributyrin plates. Afterwards recombinant expression in *Escherichia coli*, the enzyme was analyzed for its substrate specificity, optimal pH and temperature, thermal stability, and effect of different additives on its enzymatic activity. Moreover, homology modeling was performed to relate the biochemical future of the enzyme to structural properties.

## Methods

### Sampling in the marine Arctic

During two research cruises in the high Arctic samples of seawater, sediment and various biota were taken. For the sediment sampling, a Van-Veen grab was used and two 50 ml tubes of the top 10 cm layer were filled at each sampling location and frozen; first at −20 °C and later at −80 °C. Three of these sediment-samples, which are described in Table 1, were used to extract total DNA. The first sampling was conducted in the Barents Sea area in May 2010 and the second around Svalbard in October 2011.

**Table 1:** Sample description, number of positive hits and total number of screened clones

Sample number	Grain size	GPS COORDINATES		Depth (m)	Number of positive clones	Total number of screened clones
		Latitude	Longitude			
CTD241-86	Clay	N73 13.521	E16 20.547	475	11	384
CTD249-119	Clay	N77 8.920	E31 16.667	191	–	1000
HH596-1	Sand	N79 12.820	E19 18.976	0	8	2500

Both research cruises, performed by the University of Tromsø, were conducted in areas regulated by the Norwegian government, and no special sampling permission was necessary.

### High molecular weight DNA extraction and purification

The frozen sediments were aliquoted using a solid mortar pre-chilled with liquid nitrogen. A soft lysis protocol [29] was followed with some modifications. Five grams of sediment was resuspended in 10 mL of DNA extraction buffer and 100 μl of proteinase K (10 mg/ml) was added. The sample was incubated in a 56 °C water bath for 2 h with an occasional, gentle mixing. Then 1.5 mL 20 % SDS was added and samples were incubated at 60 °C for another 2 h. After centrifugation at 5000 g for 20 min, the DNA-containing supernatant was extracted with a phenol: chloroform: isoamyl alcohol mixture (25:24:1 volume ratio). Next, the aqueous phase was precipitated with isopropanol (0.7 volumes). The pellet was then washed with 70 % EtOH, air-dried and dissolved in TE buffer (pH 8.0). At this stage the raw DNA had a brown color which indicates a high content of contaminants.

To purify the DNA further, two protocols were followed; for sediment CTD 241–861 an ion-exchange hydroxyapatite column was used. Dry HTP-hydroxyapatite (Bio-Rad, USA) was resuspended in TE-phosphate buffer (10 mM Tris pH 8.0, 1 mM EDTA 25 mM Na-phosphate, pH 8.0), swirled and decanted three to four times to get rid of ultra-fine particles using a home-made HTP column in a syringe at 1000 g. The final volume of the resin was between 0.6 and 0.8 ml. The column was then equilibrated with 10 volumes of TE-phosphate buffer. The DNA solution was loaded onto the column and washed with increasing concentrations of sodium phosphate in TE-phosphate buffer (25 mM, 50 mM, 100 mM and 200 mM Na-phosphate pH 8.0). DNA was then eluted with TE-phosphate buffer containing 300 mM of Na-phosphate. Buffer exchange was performed using a Centricon 4 ml spin cartridge with a 100 kDa cut-off (Millipore, Germany). Sediment samples CTD 249–119 and HH 596–1 were purified using the Aurora DNA purifier from Boreal genomics, USA, which utilizes the SCODA (synchronous coefficient of drag alteration) DNA extraction technology [30]. Raw DNA was diluted to 5 ml in milliQ water and applied to the sample well of a precast gel cassette (1 % 0.25X TBE agarose gel, and 0.25× TBE buffer). The run parameters were as stated in the AURORA_HMW_DNA_SOIL_PROTOCOL, provided by the manufacturer.

The purified metagenomic DNA was quality checked by performing standard PCR targeting the 16S rRNA gene using universal primers (27 F and 1492R) and Taq polymerase.

### Creation of fosmid library, storage of clones and functional screening

The purified DNA was used with the Copy Control Fosmid Library Production Kit and the pCC1FOS Vector (Epicentre, USA) according to the manufacturer’s protocol, to obtain the three metagenomic fosmid libraries. Colonies were picked and grown in 400 μl LB containing 12.5 μg/ml chloramphenicol and 10 % glycerol using 1.2 ml deepwell blocks (square wells) and sealed with “breathable” film (BREATHseal, Greiner bio-one, USA). Incubation was done in a plate shaker at 37 °C and 300 rpm. After colony picking, plates were re-sealed with alumina sealing film (alumaseal, Sigma-aldrich, USA) and a lid was put on before the plates were transferred to -80 °C for storage.

For detection of esterase activity, EPI300TM-T1R *E. coli* fosmid clones were transferred to Omni trays (Thermo Scientific Nunc, USA) containing LB agar medium, 12.5 μg/ml chloramphenicol and 1 % tributyrin as synthetic substrate. The replication of fosmid libraries was made by a 96 pin library copier (Thermo Scientific Nunc, USA). The appearance of a clear halo zone around colonies within 4 days at 20 °C was considered a positive indication of esterase activity.

### Fosmid purification and sequencing

The fosmid from the clone showing strongest esterase/lipase activity (evaluated by halo size) was included together with 167 randomly selected fosmids to be sequenced. Deepwell blocks (2.2 ml square wells) containing 1.5 ml of LB medium with 12.5 μg/ml chloramphenicol, and supplemented with 1X autoinduction solution (Epicentre, USA) were inoculated with the 168 fosmid-bearing clones. The plates were incubated with shaking at 37 °C for 16 h. The fosmid DNA was then purified using the Montage 96 well kit from Millipore following the vacuum suction protocol. The resulting fosmid DNA was resuspended in 100 μl Tris buffer pH 8.0. The DNA concentration was measured using a Nanodrop Spectrophotometer at 260 nm. The concentration of DNA in each well was then adjusted to 120 ng/μl by adding more buffer. Pools of 7 × 24 fosmids were made by pipetting 4 μl of each of the 24 fosmid into 7 separate tubes. 7 individually tagged libraries were made from the pooled DNA, pooled again, and sequenced on the 454 GS-FLX machine (Roche, USA) using one half of a picotiter plate. The remaining fosmid DNA in the 96 well plates was utilized in end-sequencing by the Sanger method using BigDye chemistry and the primers T7 or EpiFOSF (forward) and EpiFOSR (reverse). All sequencing was performed at the Norwegian Sequencing Centre (NSC) in Oslo.

### Assembly and analysis of fosmid sequences

The sequence reads were screened for vector- and *E. coli* DNA and assembled using the Newbler Assembler software (454 Life Sciences), accessed remotely through the Bioportal in Oslo (now changed to Lifeportal, https://lifeportal.uio.no/). The 7 pools of sequences were separated according to their MID (Multiplex Identifier) and assembled individually. The Sanger end-sequences were then used to distinguish, within each pool, which fosmid-clone each contig originated from. This was done by local nucleotide blast searches against the assembled fosmid DNA. The complete insert belonging to the fosmid-clone showing esterase activity was further annotated and analyzed using GeneMark [31] (http://opal.biology.gatech.edu/). The GC content profile of the fosmid-DNA was analyzed online using EMBOSS Isochore with default settings (http://www.ebi.ac.uk/Tools/seqstats/emboss_isochore/). The fosmid insert containing the *lip3* gene has been deposited [GenBank: KJ538549].

### Gene cloning strategy

The *lip3* gene was amplified from purified fosmid DNA using a cloning method termed *FastCloning* [32]. The following primer pairs were used to PCR amplify pET-26b vector and insert separately:

VecFw 5′-TGTCTTAAGAGCTTACTGCACCACCACCACCACCAC -3′,

VecRv 5′-CTATCTATTATGTAATTATTCATATGTATATCTCCTTCTTAAAGTT-3′,

InsertFw 5′-AACTTTAAGAAGGAGATATACATATGAATAATTACATAATAGATAG-3′,

InsertRv 5′-GTGGTGGTGGTGGTGGTGCAGTAAGCTCTTAAGACA-3′. The expression vector encodes an in-frame C-terminal 6xHis-Tag. The PCR reaction conditions used were: 1 cycle (98 °C for 3 min), 20 cycles (98 °C 15 s, 55 °C 30 s, and 72 °C 1 min), and a final cycle at 72 °C for C 10 min. PCR reactions were performed in a MJ Research PTC 200 thermal cycler (MJ Research, Canada). *Dpn*I (Sigma-Aldrich, USA) was added to the PCR insert- and vector product separately. Vector and insert were mixed at a ratio of 1:4 and incubated for 2 h at 37 °C. The mixture was then used to transform NovaBlue Giga Singles competent cells (Novagen, Germany). The DNA sequence of the resulting construct was verified by bidirectional DNA sequencing. The expression vector containing *lip3* was then transformed into *E. coli* BL21 (DE3) cells.

### Recombinant production and purification of Lip3

*E. coli* BL21 (DE3) cells carrying pET-26b-Lip3 vector were cultivated in Luria Broth (LB) medium with 50 μg/mL kanamycin for 16 h at 37 °C. To induce protein expression, overnight culture was diluted to an OD 600 nm of 0.1 in 3-L shake flasks containing 600 ml LB medium and antibiotic (50 μg/ml kanamycin). Cultures were grown at 37 °C with an agitation rate of 140 rpm until the OD 600 nm reached 0.6. IPTG was then added to a concentration of 0.2 mM to induce the expression. The culture was incubated for a further 16 h at 20 °C. Cells were then harvested by centrifugation at 3200 *g* at 4 °C for 30 min and frozen at −20 °C. The pellet was resuspended in 50 mM Tris-HCl pH 8.0, 500 mM NaCl and 10 % glycerol, sonicated, and cleared by ultracentrifugation at 75,000 *g* for 40 min. The crude extract was filtered using a 0.45 μm membrane, and loaded on a HisTrap HP 1 ml column (GE Healthcare, England) equilibrated with 50 mM Tris-HCl pH 8.0, 500 mM NaCl, 30 mM imidazole, 10 % glycerol. Lip3 was eluted with a linear imidazole gradient (10 ml of 0–500 mM). Fractions of 1 mL were collected and analyzed by SDS-PAGE, with Lip3 being detected by the presence of a band at the expected molecular weight after Comassie staining. Fractions containing the recombinant enzyme, were dialyzed against 20 mM Tris-HCl pH 8.0, 10 mM NaCl and 5 % glycerol at 4 °C overnight.

The recombinant protein was further purified using a 1 ml HiTrap Q HP column (GE Healthcare, England) equilibrated with buffer A (20 mM Tris-HCl pH 8, 10 mM NaCl, 5 % glycerol) and eluted with a linear gradient of 0–100 % of buffer B (20 mM Tris-HCl pH 8, 1 M NaCl, 5 % glycerol) at a flow rate of 1 ml/min. The proteins containing the esterase activity eluted at approximately 50 % buffer B.

SDS-PAGE was performed using 5 % polyacrylamide-stacking gel and a 12 % polyacrylamide-resolving gel with a Bio-Rad Mini-Protean II cell unit, at room temperature essentially as described by Laemmli. Opti-Protein XL protein molecular mass marker (ABM, Canada) was used as molecular weight standard. The protein concentration was determined according to the Bradford method with bovine serum albumin as the standard [33]. The protein content was measured by monitoring the optical density at 595 nm.

### Lipolytic activity assays

The lipolytic activities of the purified enzyme were determined by measuring the hydrolysis of synthetic substrates labeled with *p*-nitrophenol (*p*-NP). The reaction progress was followed by monitoring the absorbance at 405 nm in 1-cm path-length cells with a Cary 100 spectrophotometer (Varian, Australia), equipped with a temperature controller. To check the linearity of the reaction, two different concentrations of enzyme were tested for each condition. Stock solutions of *p*-nitrophenyl (*p*-NP)-esters were prepared by dissolving the substrates in pure acetonitrile. Assays were performed in 1 mL mixture containing purified enzyme (2 μg/mL), 100 mM Tris-HCl buffer (pH 8.0), 3 % acetonitrile and *p*-NP esters at different concentrations.

One unit of esterase activity was defined as the amount of enzyme needed to release 1 μmol *p*-NP in 1 min. All experiments were performed in triplicate. Results are expressed as mean values ± SE of the mean.

### Substrate specificity and enzyme kinetics

The substrate specificity of the esterase was investigated by measuring enzymatic activity toward a series of *p*-NP esters with various carbon chain lengths: *p*-NP acetate (C2), *p*-NP butanoate (C4), *p*-NP pentanoate (C5), *p*-NP octanoate (C8), *p*-NP decanoate (C10). Assays were carried out in duplicate at 35 °C by following the absorbance at 405 nm, and the kinetic parameters were determined from the rates of hydrolysis by fitting the rates to a Lineweaver-Burk double reciprocal plot. All kinetic data were analyzed by linear regression using SigmaPlot 10.0.

### Effects of pH and temperature on Lip3 activity

Esterase activity was measured at different pHs by using the buffers 0.1 M MES (pH 5.0–6.0), 0.1 M Na-phosphate (pH 6.0–7.5), 0.1 M Tris-HCl (pH 7.5–9.5) and 0.1 M CAPS (pH 9.5–10.5). The esterase activity at 25 °C was monitored by the amount of *p*-nitrophenol released from *p*-nitrophenyl (*p*-NP) esters at 348 nm, which is the pH-independent isosbestic wavelength of *p*-nitrophenoxide and *p*-nitrophenol. A molar extinction coefficient of 5000 M^−1^ cm^−1^ at 25 °C was used in the calculations.

The activity was expressed as percent relative activity with respect to maximum activity, which was considered as 100 %.

Esterolytic activity as a function of temperature was determined in the range of 10–65 °C with 5 °C increments using *p*-NP pentanoate (100 μM) as a substrate. The reaction buffer was 0.1 M Tris-HCl (pH 8.0) as this was determined to be optimal from the pH screening, and contained 3 % acetonitrile.

### Thermal stability of the esterase

The thermostability of the enzyme was examined at temperatures ranging from 25 to 70 °C. The enzyme was incubated at different temperatures for a total time of 2 h, and the residual activity was measured at 20-min intervals under standard conditions.

### Effect of additives and NaCl on enzyme activity

The effect of various additives on esterase activity was tested by incubating the protein for 1 h at 4 °C in presence of β-mercaptoethanol, EDTA, DTT or PMSF at final concentrations of 1 mM and 10 mM. The residual activities were measured by comparison with activity from the standard assay, containing no compounds, and defined as 100 %.

The effect of NaCl on esterase activity was investigated by assaying with increased salt concentrations in a range from 0 to 4 M at 35 °C using standard assay conditions. Additionally, assays were carried out after incubation of the enzyme in presence of 0, 1, 2, 3 M NaCl for 24 h at 4 °C.

### Sequence analysis

The Lip3 sequence was investigated for protein homology by searching the complete non-redundant protein databases (www.ncbi.nlm.nih.gov) using the BLAST software [34]. A multiple sequence alignment was constructed using the JalView software (Fig. 6) [35]. The N-terminal signal peptide prediction was made using SignalP 3.0 (http://www.cbs.dtu.dk/services/SignalP/) [36]. Molecular weights were determined using Protein Calculator V3.3 (http://www.scripps.edu/~cdputnam/protcalc.html).

### Lip3 modeling

Lip3 was modeled using four templates, obtained by scanning the Protein Data Bank database with the HHpred server [37]: a triacylglycerol lipase from *Yarrowia lipolytica* [PDB: 3O0D], a lipase from *Gibberella zeae* [PDB: 3NGM], a lipase from *Penicillium expansum* [PDB: 3G7N] and a lipase from *Serratia marcescens* [PDB: 2QUB]. The atomic coordinates of the templates were obtained from the Protein Data Bank. In order to create the 3D-model, the multiple sequence alignment between Lip3 and the sequences of the four templates, obtained in PIR format by HHpred server [32], was submitted to the comparative protein structure modeling software Modeller 9v11 [38]. Modeller algorithm was set to generate 53-dimensional models. To select the best model, structure validation was carried out by PDBsum pictorial database. In order to evaluate the stereochemical quality of the generated structures, models were uploaded, in the standard PDB file format, to the PDBsum server, to carry out a full set of Procheck structural analyses [39]. Moreover, the Z-score of the Lip3 model was calculated using the WhatIf web server [40]. The Z-score expresses how well the backbone conformations of all residues correspond to the known allowed areas in the Ramachandran plot. The Solvent Accessible Surface Area (SASA) of the templates and Lip3 model was calculated by POPS algorithm [41]. Furthermore the electrostatic potential of Lip3 external surface was performed by APBS (Adaptive Poisson-Boltzmann Solver) and PDB2PQR software packages [42, 43]. Finally the molecular graphics software VMD [44] was used to display the obtained model (Fig. 7).

## Results

### Construction of metagenomic libraries and screening for lipolytic enzymes

Three small fosmid libraries were created from marine sediment samples. The total number of fosmid clones and the number of positive hits in the screening are shown in Table 1, together with the exact GPS coordinates.

**Fig. 1:**
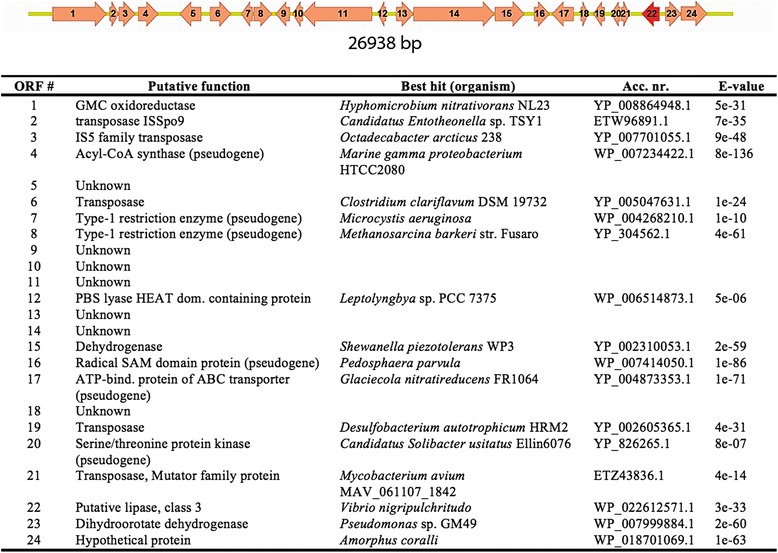
Arrangement of open reading frames (ORFs) encoded by the fosmid-insert. The Lip3 encoding gene is marked in red. Table shows the predicted function of the ORFs in the lip3-fosmid predicted by GeneMark

A total of 19 fosmid clones showed a clear halo zone indicative of putative lipolytic activity (see Additional file 1). The clone displaying the largest halo size was sequenced, and a gene encoding a putative class 3 lipase was found. As shown in Fig. 1, we could not detect any clear phylogenetic relatedness of the ORFs encoded by the fosmid. Each ORF is similar to a variety of unrelated bacteria and no phylogenetic marker gene is present. The presence of four transposase genes in the fosmid indicates that the region which is cloned comes from a region within the host DNA with rearrangements and/or insertions due to the transposases. This is consistent with its highly variable GC-content profile (data not shown). An 843-bp ORF encoding a putative esterase/lipase (designated Lip3) was identified by GeneMark. The low sequence conservation, between Lip3 and the eight lipolytic families described by Arpigny [13], did not allow the construction of a meaningful phylogenetic tree for the full dataset. Despite this a sequence analysis using the Pfam protein families identified a α/β-hydrolase family sequence (Pfam01764) belonging to class 3 lipases. The lipase catalytic triad (serine, aspartate and histidine) is indicated (Fig. 7). Lip3 contain no signal peptide as predicted by SignalP.

### Expression and purification of recombinant Lip3

In order to study the biochemical properties of the enzyme, the *lip3* gene was cloned into pET26b in frame with the C-terminal 6x His tag encoded by the vector.

High amounts of active protein were achieved when *E. coli* BL21 (DE3) was induced overnight with 0.2 mM IPTG at 20 °C. The expressed protein was purified to homogeneity with a yield of 1.5 mg of protein from 2 L of cell culture and a SDS-PAGE analysis shows that under denaturing condition the molecular weight (MW) is around 31.2 kDa (see Additional file 2).

### Effect of pH and temperature on enzyme activity and stability

**Fig. 2:**
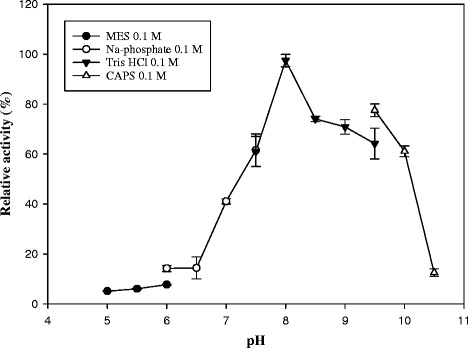
Effect of pH on Lip3 activity. Relative activity of *p*-NP-pentanoate (100 μM) hydrolysis was performed in various pH buffers at 25 °C

The effect of pH on esterase activity was assessed at 25 °C (Fig. 2) using *p*-NP-C5 as substrate. The enzyme in the pH range of 5.0–10.5 showed maximal activity at pH 8.0 in Tris-HCl buffer.

**Fig. 3:**
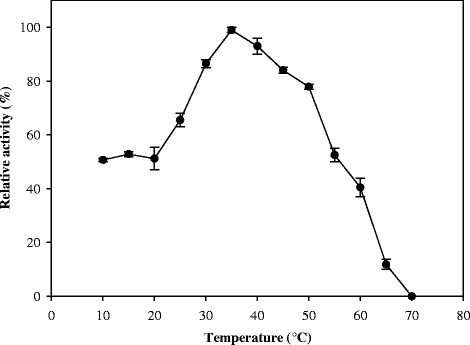
Effect of temperature on Lip3 esterase activity. The activity was determined at different temperatures in 0.1 M Tris-HCl buffer adjusted to pH 8.0 using *p*-NP-pentanoate (100 μM) as substrate

The effect of temperature on esterase activity was determined using *p*-NP-C5 as substrate. Lip3 was active over a temperature range from 7 to 65 °C (Fig. 3), with an optimum temperature of 35 °C. No catalytic activity was detectable at 70 °C.

Lip3 hydrolysed *p*-NP esters with acyl-chain lengths from two to ten carbon atoms (C2–C10). All characterization was performed at 35 °C and in presence of 3 % acetonitrile using different stock solution in a range of 7-50 mM. In this condition, the enzyme displays highest activity towards *p*-NP acetate (Table 2) with a highest *k*_*cat*_ and *k*_*cat*_*/K*_*M*_ value. Enzymatic activity was assayed in triplicate with an appropriate blank for the correction of the auto hydrolysis of the substrate. This result showed that the enzyme is an esterase and *p*-NP acetate was used as its preferred substrate for further studies.

**Table 2:** Kinetic parameters for Lip3

Substrate	*k*_cat_ (s^−1^)	*K*_M_ (M∙10^−3^)	*s = k*_cat_ / *K*_M_ (sec^−1^∙M^−1^∙10^3^)
*p*NP-acetate	1198 ± 200	2.38 ± 0.56	503.3 ± 214.5
*p*NP-butanoate	218.0 ± 15.5	1.05 ± 0.16	206.5 ± 48.6
*p*NP-pentanoate	152.8 ± 9.82	1.52 ± 0.20	100.2 ± 19.7
*p*NP-octanoate	100.0 ± 3.8	0.36 ± 0.04	271.0 ± 44.0
*p*NP-decanoate	29.9 ± 1.5	1.03 ± 0.12	29.3 ± 10.1

Esterase activity was measured at 35 °C for five minutes in presence of 0.1 M Tris-HCl pH 8.0

**Fig. 4:**
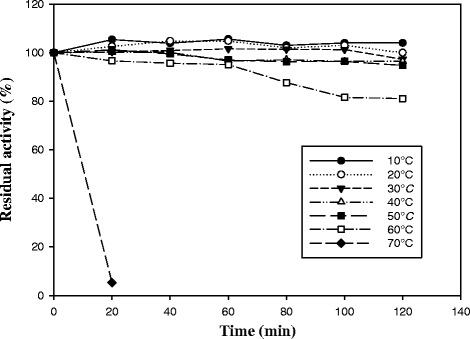
Thermal stability of Lip3 esterase. Activity was measured in the range from 10–70 °C using *p-*NP-acetate (100 μM) as substrate

To examine the thermal stability of Lip3 esterase, we pre-incubated the enzyme at different temperatures and measured the residual activity under standard assay conditions. The enzyme displayed a relatively high thermal stability at 60 °C, retaining approximately 80 % of its activity even after incubation for 120 min (Fig. 4). However, the stability of the enzyme decreased significantly after only 20 min at 70 °C.

### Lip3 activity in presence of additives and NaCl

The enzyme was unaffected by the presence of low concentrations of β-mercaptoethanol and DTT, while low concentrations of PMSF and EDTA gave a small but detectable decrease in activity. The relative activity was approximately halved when the additive concentration was tenfold higher (Table 3).

**Table 3:** Effect of different additives on Lip3 activity

	Relative activity %	
Additives	Concentration	Concentration
	1 mM	10 mM
Control	100 ± 0.007	100 ± 0.004
β-mercaptoethanol	96.0 ± 0.005	56.0 ± 0.002
EDTA	91.0 ± 0.001	78.7 ± 0.003
DTT	106.0 ± 0.021	59.0 ± 0.005
PMSF	77.0 ± 0.011	55.0 ± 0.004

Esterase activities were measured toward various compounds at 35 °C in presence of 0.1 M Tris-HCl pH 8.0

**Fig. 5:**
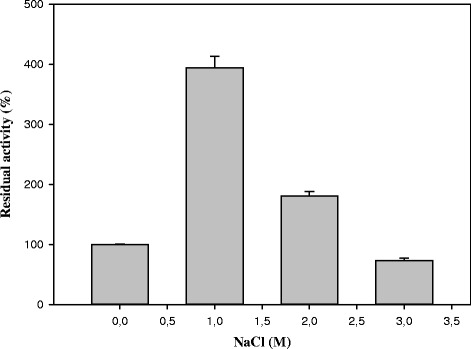
Stability profile of Lip3 with NaCl. Lip3 activity was evaluated after preincubation in presence of NaCl at 4 °C for 24 h

Lip3 activity was evaluated in presence of NaCl under the above mentioned assay conditions in 0.1 M Tris-HCl pH 8.0. Results show the activating effect of NaCl on Lip3, with the highest activity value being obtained in 3 M NaCl (Table 4). To test the stability of the enzyme with increasing amount of NaCl we measured the relative activity after incubation for 24 h at 4 °C. The stability approximately increased by a factor of four in presence of 3 M NaCl (Fig. 5) and a factor of approximately two in presence of 2 M NaCl.

**Table 4:** Effect of NaCl on Lip3 activity

NaCl (M)	Relative activity (%)
0	100.0 ± 0.093
1	284.3 ± 0.107
2	334.3 ± 0.003
3	675.0 ± 0.021
4	528.0 ± 0.007

Esterase activity was measured at different NaCl concentrations using *p-*NP-acetate as substrate

### Analysis of the Lip3 sequence

**Fig. 6:**
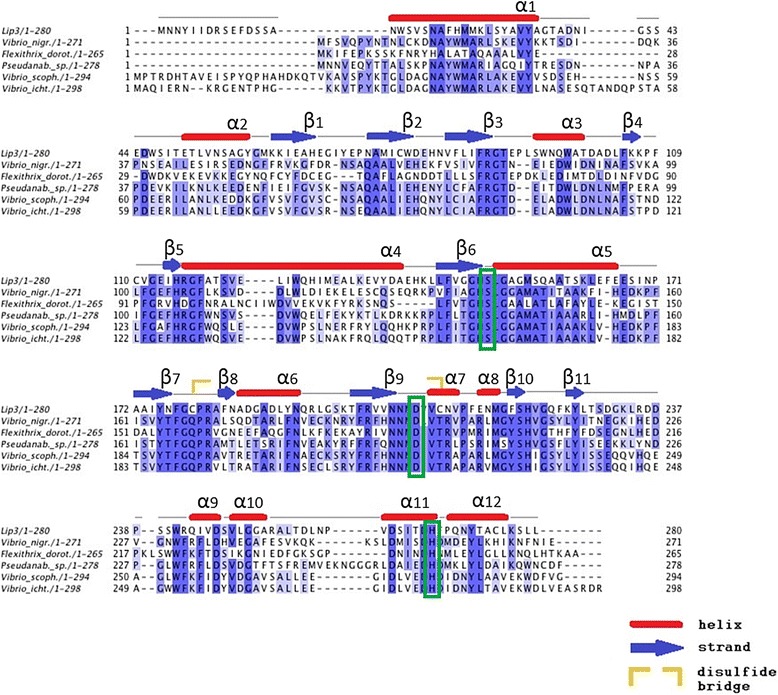
Multiple alignment of Lip3 and the most similar sequences found using BLASTp against the non-redundant protein database. The most conserved residues are shown in dark blue. The catalytic triad is highlighted by green rectangles. Secondary structure elements obtained from the modeling of Lip3 are as indicated

A multiple sequence alignment, consisting of Lip3 together with the most similar amino acid sequences is shown in Fig. 6. Similarities were found with lipases from *Vibrio scophthalmi* and *Vibrio ichthyoenteri*, hypothetical proteins from *Flexthrix Dorotheae* and *Pseudanabaena sp.* PCC 6802, and a putative lipase from *Vibrio nigripulcritudo*. These sequences share only about 30 % identity and 50 % similarity with the Lip3 sequence. The alignment (Fig. 6) reveals that Lip3 contains the lipase-conserved catalytic triad residues, Asp207, His267 and the catalytic nucleophile Ser150, in the typical consensus pentapeptide G-X-S-X-G, also known the nucleophilic elbow.

### Lip3 modeling

**Fig. 7:**
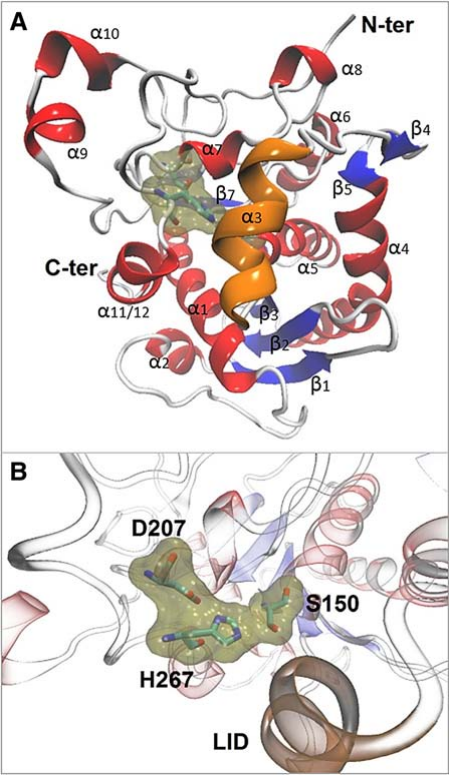
**a** 3D-model of Lip3. Helices are shown in red. Strands are shown in blue. The one-helix lid is shown in orange. The catalytic triad is shown in yellow. **b** Close up view of Lip3 catalytic triad. The residues forming the catalytic triad are shown in yellow. The one helix lid is shown in orange

The 3D-modeling of Lip3 was performed by homology modeling using the following structures as templates: a triacylglycerol lipase from *Yarrowia lipolytica* [PDB ID: 3O0D], a lipase from *Gibberella zeae* [PDB: 3NGM], a lipase from *Penicillium expansum* [PDB: 3G7N] and a lipase from *Serratia marcescens* [PDB: 2QUB]. These structures were chosen because they were predicted to have a significant structural homology with Lip3, calculated by HHpred server, despite a low percentage of sequence identity. Starting from the alignment of Lip3 sequence with the reference structures, a set of 50 all atoms models was generated. The best model (Fig. 7a) was selected in terms of energetic and stereochemical quality. In detail, it has 86.3 % of residues in the most favored regions and no residues in disallowed regions of the Ramachandran plot according to the PROCHECK program provided with PDBSum. Moreover this model has a WhatIf Z-score of −0.297, which is within expected ranges for well-refined structures. These values, compared with those of the template structures, indicated that a good quality model was created. The Lip3 model displayed an alpha-beta structure characterized by 9 alpha-helices, three 310-helices and 11 beta-strands forming three sheets (Fig. 7), corresponding to 35, 5.4 and 16.8 % of sequence, respectively. Moreover the Lip3 structure seems to be stabilized by a disulfide bond (Cys179-Cys210). The SASA analysis of the Lip3 model carried out by the POPS algorithm revealed an exposed surface that is more hydrophobic than hydrophilic (59.6 % versus 40.4 %, see Additional file 3). On the contrary, the SASA analysis of the template structures always revealed an exposed surface that is less hydrophobic than hydrophilic (38.5 % versus 61.5 % for the *Penicillium expansum* lipase, 32.7 % versus 67.3 % for the *Gibberella zeae* lipase, 34.3 % versus 65.7 % for the *Yarrowia lipolytica* triacylglycerol lipase, 35.7 % versus 64.3 % for the *Serratia marcescens* lipase). Finally the analysis of the electrostatic potential of the external surface of Lip3 revealed the presence of positive and negative charged areas (see Additional file 3) around the active site.

## Discussion

In the current study we have identified a cold-active esterase (Lip3) originating from marine clay sediment, as result of functional screening of three small fosmid libraries. The choice of sampling area was based on the knowledge that marine habitats represent a vast resource of novel lipolytic genes due to the numerous microorganisms living there. Lipids from phytoplankton are one of the principal nutrition sources in marine food chains [45] and these can be removed from the surface layers by microbial activity when falling through the water column and lying on the benthos [46, 47]. The number of clones positive for esterase activity was very high for sample number 1 (11 out of 384). We do not have an explanation for this, however the sample sites were not carefully inspected, and there are probably great variations of nutrients available at the respective sites. Additionally, the occurrence of false positives is a well-known phenomenon when using tributyrin as substrate [48]. As we did not sequence more of the positive fosmids, we can only speculate the reasons for this high hit-rate.

To address the biotechnological potential of Lip3 as a biocatalyst, we performed a biochemical and structural characterization of the recombinant enzyme. Lip3 showed activity at temperatures as low as 10 °C and has an optimum temperature of 35 °C, so it can be defined as a cold-active esterase. The highest relative activity was observed at pH 8.0 while a loss of activity was observed at pHs below 7.0 as reported for other identified esterases [49]. The sudden drop in activity at acidic pH can be explained by deprotonation of His267 (pKa 6.08). The drop at alkaline pH could be due to the protein denaturation rather than protonation changes in the active site residues.

The substrate specificity experiment revealed that in vitro the enzyme functions best as an esterase with a preference for *p*-NP esters of short- and medium chain fatty acids and could not hydrolyze substrates with long-chain fatty acids. In agreement with our results, another esterase from sea sediment metagenome was also found to have the highest hydrolytic activity with short- and middle- length *p*-NP esters [24].

In addition, Lip3 activity was evaluated in the presence of DTT, EDTA, β-mercaptoethanol and PMSF, at low and high concentrations. An inhibitory effect was observed at high concentrations additives. The inhibition of esterase activity in the presence of EDTA can be attributed to its metal chelating effect. The weak inhibition of esterase activity caused by PMSF may be attributed to the attack of Ser150 responsible for the hydrolytic reaction in the active site since this inhibitor interacts selectively and irreversibly with the serine hydroxyl groups [50]. The relatively low reactivity could be explained by the low stability of PMSF in the assay conditions. A hypothetical disulfide bond between Cys179 and Cys210 was seen in the model, and the high concentrations of DTT and β-mercaptoethanol probably reduce this bond, thereby lowering the enzyme activity. Being an intracellular enzyme, the disulfide bonds rarely form due to the reducing environment. The surface exposure of the disulfide bond might be low, so that it is more shielded from being naturally reduced.

The enzyme thermostability studies showed a quite stable behavior up to 60 °C for 1 h which decreased at higher temperatures. This is in contrast to the stability of a previously published cold-active lipase EstF, from deep-sea metagenomic library, which was stable up to 50 °C and had a dramatic decrease thereafter [51].

The Lip3 enzyme thus shows stability at moderate temperatures, and because of its high catalytic efficiency and specificity at low and moderate temperatures, it could be used in improving biotechnological or industrial processes. Applications may include the use of the enzyme as catalyst for organic synthesis of unstable compounds at low temperature [52]. Due to the high stability in presence of NaCl, Lip3 could also be used in food technology applications [53] to accelerate cheese flavor during ripening. The advantage is to use this enzyme at the ripening temperature (7 to 53 °C) followed by an easy inactivation at higher temperatures to avoid the development of strong rancid flavor. Lip3 may be added individually or as a cocktail with other lipases.

Structural analysis was carried out focusing on the most conserved regions in the sequence alignment (Fig. 6). In Fig. 7b, the catalytic triad of Lip3, formed by Ser150, Asp207 and His267, is highlighted in yellow. The three amino acids forming the catalytic triad seem to be located in three different loops connecting three strands to three helices. In particular Ser150 is within a conserved motif called a “nucleophilic elbow”.

The solvent accessible surface area analysis suggests that this enzyme, thanks to its prevailing hydrophobic surface (59.6 %), could be adapted to hydrophobic environments, such as organic solvents, despite the opposite trend shown by the 4 templates (38.5 %, 32.7 %, 34.3 %, 35.7 %). In particular, the active site is covered by a mobile element, the lid (Fig. 7a), which opens when the enzyme binds a hydrophobic interface [54]. The surface exposed to the active site is hydrophobic, whereas the surface exposed to the outside of the enzyme is hydrophilic [55–57]. When the lid opens and exposes its hydrophobic surface, the SASA (solvent-accessible surface area) increases drastically [58]. Depending on the structure of the lid, different transition mechanisms have been proposed. In lipases with a simple one-helix lid, it is assumed that the transition is a fast rigid body movement [59]. In lipases with a more complex lid, instead, the secondary structure of this mobile element changes when it opens, undergoing a partial refolding [52] which might be a kinetic bottleneck [60]. Lip3 seems to belong to the first category, having a single-helix lid (Fig. 7a, α3) and it shows a higher catalytic activity if compared to other kinetic values of esterases and lipases described in the literature [61, 62].

It is already known that salt has a significant impact on the protein stability [63]. In our results, as shown in Fig. 5 and in the additional file 3, hydrate ions can have interacted with surface residues to stabilize the folded Lip3 conformation in presence of high NaCl concentrations. The theoretical isoelectric point (pI) of Lip3 is 4.98 which means that the global negative net charge of the esterase would be negative in a buffer solution of pH 8.0. Under these conditions, the substrates *p*-nitrophenyl esters are positively charged. The increase of the activity at high salt concentrations might be due to a salt effect on the hydrophobic interaction between the active site of Lip3 and the substrate. The decrease of Lip3 activity at really high salt concentrations might be due to a screening effect by salt of charge-charge amino acids interactions [64].

Crystallization of Lip3 will clearly be of value in future studies. Afterwards, structural studies of docking interactions between Lip3 and natural fat substrates will be a key to develop an improved mutant esterase which can ideally be extensively used in the dairy industry.

## Conclusions

In summary, a novel cold-active esterase, Lip3, was isolated from a metagenomic library constructed from an Arctic marine sediment sample. A homology model of the enzyme highlighted the presence of a mobile element (lid) covering the active site consisting in a catalytic triad (Ser150, Asp207 and His267). Moreover the 3D-model, despite the presence of charged areas in the external surface, revealed a surface area more hydrophobic than hydrophilic. Lip3 showed high activity both at low temperatures and in presence of high salt concentrations. These are very useful characteristics for biotechnological processes. The described esterase with its characteristics is a valuable contribution to the expanding enzymatic toolbox used by academia and the biotechnological industry.

## Availability of supporting data

The data sets supporting the results of this article are included within the article and three additional files.

## Additional files

Additional file 1:
**Functional screening of a fosmid library on 1 % tributyrin (glycerol tributyrate) agar plates incubated at 20 °C.** A clear halo zone is indicative of putative lipolytic activity. (PNG 480 kb) Additional file 2:
**SDS-PAGE analysis of the purified recombinant esterase, Lip3.** Lane 1, purified esterase, Lip3 (31.2 kDa); Lane 2, Opti-Protein XL protein molecular mass marker. (PNG 224 kb) Additional file 3:
**Graphical representations of the electrostatic potential in the external surface of Lip3 model.** Positive charges are shown in blue. Negative charges are shown in red. Uncharged areas are shown in white. Catalytic triad cloud is shown in yellow. (PNG 1699 kb)

## Abbreviations

DTT: Dithiothreitol
EDTA: Ethylene-Diamine-Tetra-Acetic acid
EtOH: ethanol
IPTG: Isopropyl β-D-1-thiogalactopyranoside
LB: Luria-Bertani
MW: Molecular Weight
OD: optical density
PCR: polymerase chain reaction
PDB ID: Protein Data Bank Identifier
PMSF: Phenylmethanesulfonylfluoride
pNP: *para* -Nitrophenol
RCF: relative centrifugal force
SASA: Solvent Accessible Surface Area
SDS-PAGE: Sodium dodecyl sulfate - polyacrylamide gel electrophoresis
TE: Tris EDTA
