# Supplementary figures and images for: Characterization of a cold-active and salt tolerant esterase identified by functional screening of Arctic metagenomic libraries
Source: BMC Biochem. 2016 Jan 19;17:1. doi: 10.1186/s12858-016-0057-x (PMC4717575; doi:10.1186/s12858-016-0057-x)

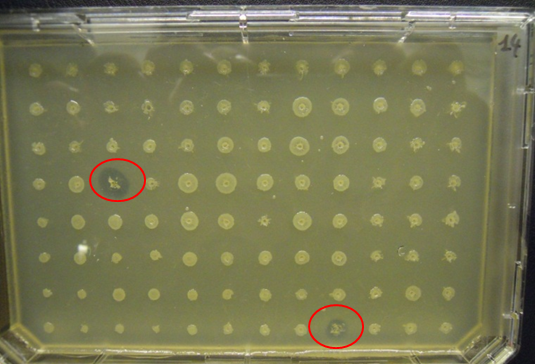

Supplement: Additional file 1: — Functional screening of a fosmid library on 1 % tributyrin (glycerol tributyrate) agar plates incubated at 20 °C. A clear halo zone is indicative of putative lipolytic activity. (PNG 480 kb) [file 12858_2016_57_MOESM1_ESM.png]

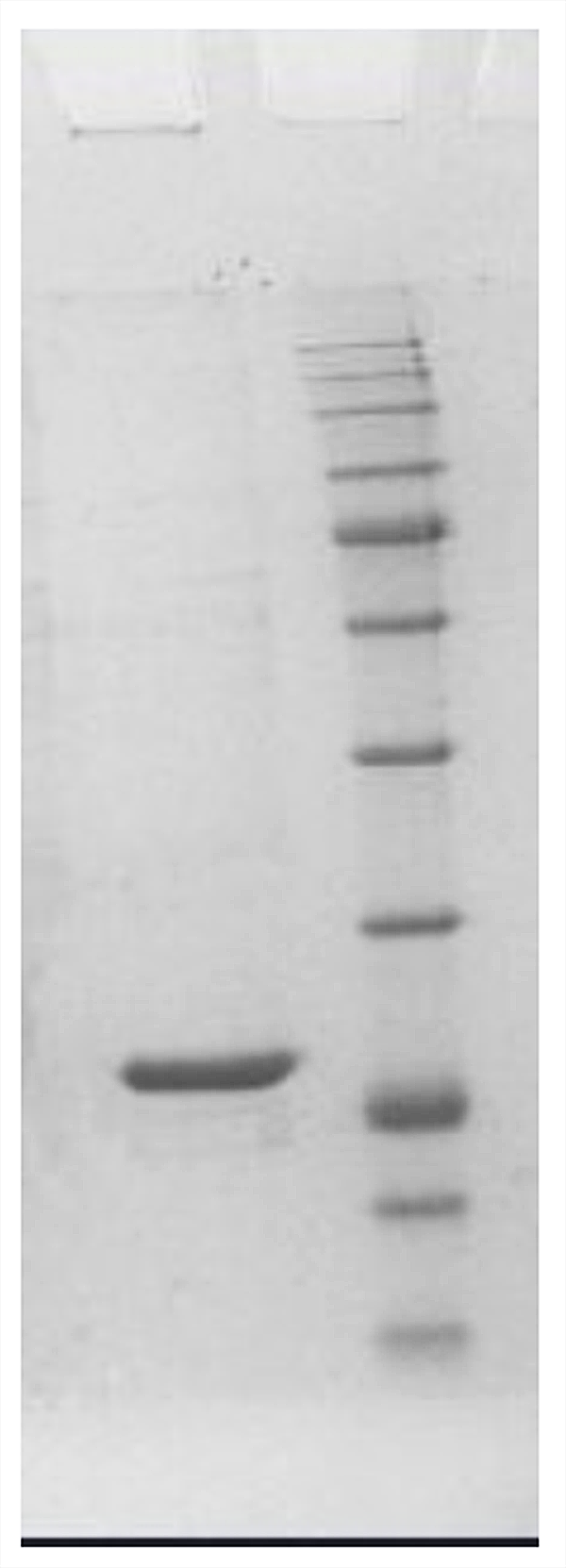

Supplement: Additional file 2: — SDS-PAGE analysis of the purified recombinant esterase, Lip3. Lane 1, purified esterase, Lip3 (31.2 kDa); Lane 2, Opti-Protein XL protein molecular mass marker. (PNG 224 kb) [file 12858_2016_57_MOESM2_ESM.png]

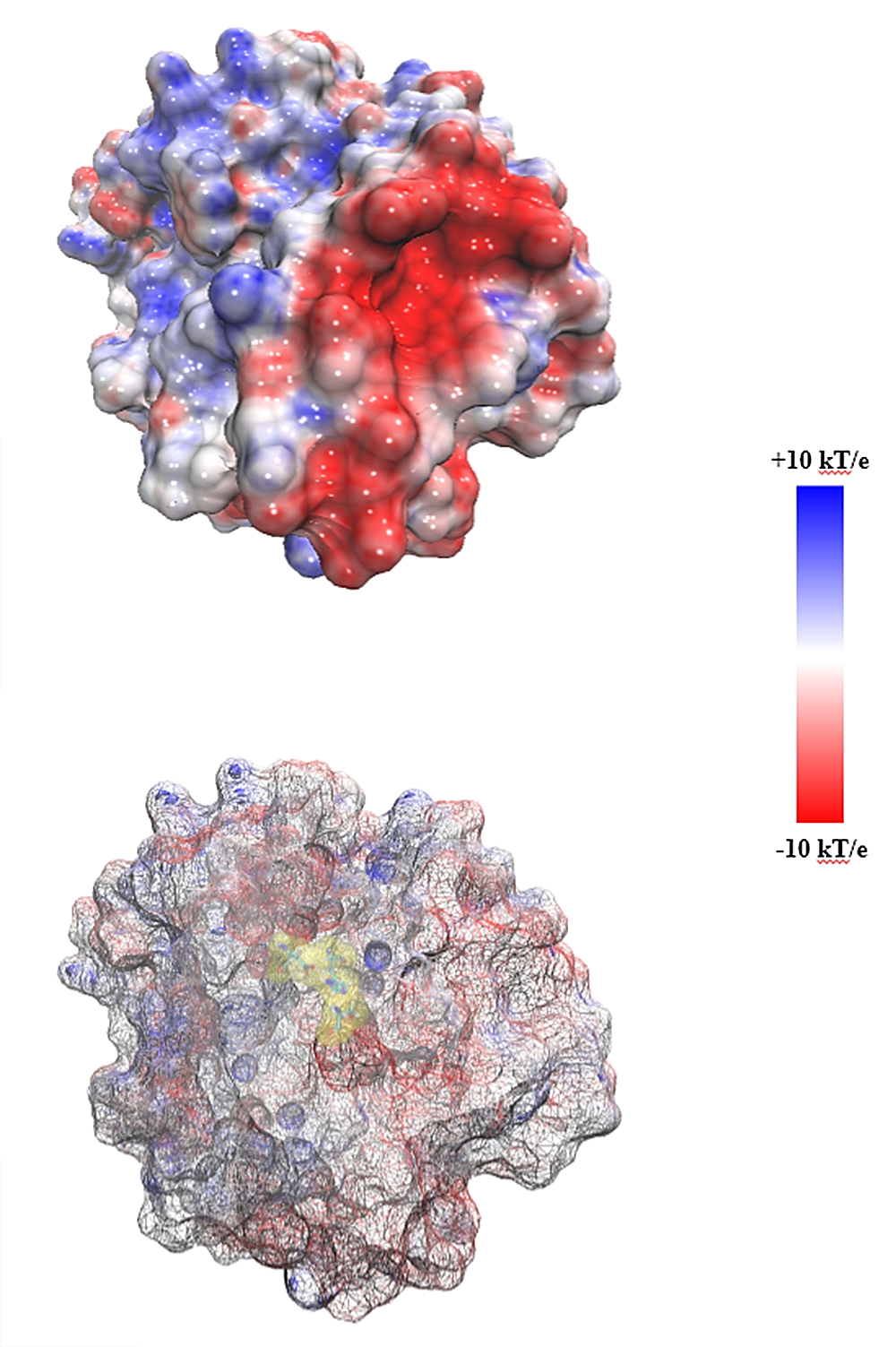

Supplement: Additional file 3: — Graphical representations of the electrostatic potential in the external surface of Lip3 model. Positive charges are shown in blue. Negative charges are shown in red. Uncharged areas are shown in white. Catalytic triad cloud is shown in yellow. (PNG 1699 kb) [file 12858_2016_57_MOESM3_ESM.png]
